# Senescence and autophagy in usual interstitial pneumonia of different etiology

**DOI:** 10.1007/s00428-020-02917-2

**Published:** 2020-08-27

**Authors:** Florian Gallob, Luka Brcic, Sylvia Eidenhammer, Florian Rumpp, Andreas Nerlich, Helmut Popper

**Affiliations:** 1 Diagnostic and Research Institute of Pathology, Medical University of Graz, Neue Stiftingtalstrasse 6, 8010 Graz, Austria; 2 Pulmonology Department, Clinics Munich-Bogenhausen, Munich, Germany; 3 Pathology Department, Clinics Munich-Bogenhausen, Munich, Germany

**Keywords:** IPF, UIP, Senescence, p16, Autophagy, LC3, SIRT, MAP1S, pAMKα, Regeneration in cysts, Cytokeratin 5, TTF1, Myofibroblast, Hypoxia, LDH, GLUT1

## Abstract

Idiopathic pulmonary fibrosis (IPF) is a disease with a dismal prognosis. Currently, the causing agent(s) are poorly understood. Recent data suggest that senescence and autophagy might play a role in its development, as well as changes in metabolism due to hypoxic conditions. In this study, the expression of senescence markers in 23 cases of usual interstitial pneumonia (UIP)/IPF and UIP/chronic autoimmune diseases (UIP/AuD) was investigated. The status of autophagy was evaluated with respect to either antiinflammatory or antihypoxia function. Formalin-fixed paraffin-embedded tissues of UIP were selected for immunohistochemistry with antibodies for p21, p16, and β-galactosidase (senescence); for LC3, SIRT1, MAP1S, and pAMKα (autophagy); and for LDH and GLUT1 (metabolism). Epithelial cells in cystic remodeled areas of UIP stained for p16 and p21, p16 being more specific compared with p21. Myofibroblasts were negative in all cases. An upregulation of all four autophagy markers was seen not only in epithelia within remodeled areas and proliferating myofibroblasts, but also in bronchial epithelia and pneumocytes. Upregulated autophagy points to a compensatory mechanism for hypoxia; therefore, LDH and GLUT1 were investigated. Their expression was present in epithelia within cystic remodeling and in myofibroblasts. The cells within the remodeled areas stained for cytokeratin 5, but coexpressed TTF1, confirming their origin from basal cells of bronchioles. Within this population, senescent cells arise. Our results indicated that autophagy in UIP very likely helps cells to survive in hypoxic condition. By phagocytosis of cellular debris, they supplement their need for nutrition, and by upregulating LDH and GLUT1, they compensate for local hypoxia.

## Introduction

Interstitial lung diseases (ILDs) are a heterogenous group of diseases with different etiology, clinical, radiological, and histologic presentation. They can be classified based on etiology (known and unknown) or based on histology, with usual interstitial pneumonia (UIP) as the most common pattern [24, 44, 49, 60]. Idiopathic pulmonary fibrosis (IPF) is the most severe disease characterized by an UIP pattern. Many aspects of the pathogenesis have been reported, such as premature aging of pneumocytes due to defects in the telomere or the surfactant gene system [15, 30, 31, 36, 42, 46, 47, 53, 55]. Initially, the occurrence of UIP pattern in diseases other than IPF was not accepted; however, this changed. Furthermore, it was recognized that the disease course in chronic autoimmune diseases with UIP pattern (UIP/AuD) has a similar dismal course as IPF (UIP/IPF) [1]. The incidence of IPF in Europe ranges between 5 and 8.5 per 100,000 individuals per year with short mean survival of 2.5 to 5 years after diagnosis [21, 40]. Current therapy includes deceleration of the disease progression with antifibrotic drugs Nintedanib and Pirfenidon, symptom release with oxygen, and, as ultima ratio, lung transplantation [13, 32]. Two processes might play a key role in development of UIP/IPF: senescence and autophagy [37, 45].

Senescence is the state of cell cycle arrest, which is initiated by cyclin-dependent kinase inhibitors such as p16, p21, and p53. It is caused by DNA damage and is usually age related [54]. Senescent cells secrete various mediators (e.g., IL-1, IL-6, IL-10, TGF-β), which promote fibrosis and may play a role in the epithelial mesenchymal transition in UIP, which may be brought on by dysfunctional autophagy [6, 8, 38, 45, 52, 53]. Senescent cells can be identified by the surrogate markers β-galactosidase, and p21 and p16, inducing senescence through cell cycle inhibition [11, 16, 20, 27, 34]. These markers may be used for immunohistochemical detection [41]. Autophagy is a cellular process for degradation and recycling of cellular debris, important for homeostasis. It can downregulate inflammation, thus inhibiting the action of senescent cells [7, 23, 58]. Under specific conditions, such us hypoxia, starvation, or the absence of growth factors, autophagy is considerably increased [26]. It was observed that markers for autophagy (LC3-II and the number of autophagosomes) are reduced in IPF lung cell lysates, indicating that dysfunctional autophagy might lead to senescence and myofibroblast trans-differentiation into epithelial cells [5, 43]. However, more recent studies demonstrated that autophagy was necessary for TGF-β-induced fibrosis in UIP/IPF and that markers for autophagy are seen in both epithelial and mesenchymal cells, whereas samples from UIP/AuD donors showed less autophagic activity [17]. Autophagic activity can be demonstrated using antibodies for adenosin-5′ monophosphate-activated kinase (AMPK), known as an activator of autophagy, or for microtubule-associated protein 1A/1B-light chain 3 (LC3) and microtubule-associated protein 1S (MAP1S), both involved in the development and degradation of autophagosomes [18, 43, 51, 63]. Nuclear protein SIRT1, a member of the silent information regulator (SIR) gene family, takes part in DNA damage prevention and repair and induces autophagy, and might also be used as a marker of autophagy [25]. Yet, there are controversial studies about the protective effect of SIRT1 against senescence, some stating that SIRT1 may even reverse senescence; other observed that continuous SIRT1 stimulation leads to irreversible senescence [33, 56].

Epithelial-mesenchymal transition (EMT) has also a role in the development of UIP/IPF. However, the exact origin of the cells replacing damaged pneumocytes in UIP is not known. One theory is that basal cells from the upper airways, which have stem cell properties, migrate to remodeled areas in UIP [39, 61]. The staining pattern of cytokeratin 5 (CK5) may be one way to investigate the origin of cells replacing the damaged pneumocytes in UIP. CK5, physiologically expressed by basal cells in upper airways, was observed in the epithelia of remodeled areas in UIP samples, and was also pronounced in the distal airways and alveoli when compared with healthy samples [48, 64]. Staining for the thyroid transcription factor 1 (TTF1) will additionally point towards the origin of these epithelial cells from peripheral lung epithelia [57]. The origin of myofibroblasts in UIP is not known. Some theories prefer EMT; other suggest involvement of circulating mesenchymal precursor cells from the bone marrow [3]. Another important factor for the induction of myofibroblast differentiation, and therefore for the pathogenesis of lung fibrosis, is lactic acid. It was shown that lactate-dehydrogenase-A (LDH-A), the enzyme which produces lactate, is overexpressed in UIP, and a decrease in extracellular pH induces a rise in TGF-β, a known mediator of pulmonary fibrosis [22, 28]. Therefore, the dysregulation of cellular metabolism might be a key factor in the genesis of lung fibrosis. However, how GLUT1 is connected to this dysregulation and the associated hypoxia is not clear. In normal lungs, GLUT1 is expressed only in erythrocytes. In UIP, some state that in areas of fibrosis, fibroblasts induce increased levels of GLUT1-dependent glycolysis to compensate for an elevated energy demand [9]. On the contrary, others state that overexpression of GLUT1 in UIP/IPF is not due to expression in fibroblasts, but in erythrocytes and inflammatory cells (macrophages), probably caused by increased neovascularization and inflammation [12].

The aim of our study was to determine the presence of senescence cells in UIP and to evaluate if autophagy is upregulated or downregulated. Downregulation of autophagy might counteract senescence by removal of cellular debris, whereas upregulation might point to a mechanism of protecting senescence cells from hypoxic stress. This might lead to a connection with metabolism under hypoxic condition in UIP. The final question was where the cells in remodeled areas (including senescent cells) originate.

## Material and methods

### Study population and clinical data

During the period from 2012 to 2019, 12 cases from the Hospital München-Bogenhausen and the Lung Archive of the Diagnostic and Research Institute of Pathology, Medical University of Graz, were selected based on pathologic reports and if a definite clinical diagnosis was available. There were 10 cases of IPF/UIP and 13 of UIP due to chronic autoimmune diseases (UIP/AuD) (Table 1). All slides were re-evaluated by two of the authors (HP, LB). The criteria for UIP were focal fibrosis, myofibroblastic foci, temporal heterogeneity (normal lung lobules, myofibroblastic foci, fibrosis), geographical heterogeneity (peripheral accentuation—only in cases with VATS biopsies), and cystic remodeling with bronchiolar metaplasia.

**Table 1:** Clinical and radiological data of patients; all patients had definite UIP by histology. *ILD* interstitial lung disease, *HP* hypersensitivity pneumonia

Age	Gender	Smoking history	CT scan	Clinical diagnosis (working hypothesis)	Final diagnosis including histology
51	F	No	ILD, NSIP?	Undifferentiated collagen vascular disease	Systemic sclerosis
82	M	Yes	UIP	Collagen vascular disease	Rheumatoid arthritis
78	F	No	UIP	Systemic disease	Rheumatoid arthritis
72	M	No	ILD	Systemic disease	Rheumatoid arthritis
74	M	No	IPF	Did not fit to IPF	Systemic sclerosis
51	M	No	UIP/IPF	Collagen vascular disease	Rheumatoid arthritis
69	M	Yes	ILD	Mixed collagen vascular disease	Mixed collagen vascular disease
57	F	No	ILD	Interstitial lung disease of unknown cause	Systemic sclerosis
50	M	No	ILD	Interstitial lung disease of unknown cause	Mixed collagen vascular disease
75	M	Yes	HP	Do not fit into HP	Chronic autoimmune disease
73	M	Yes	HP	Do not fit into HP	Chronic autoimmune disease
73	M	No	ILD	Interstitial lung disease of unknown cause	Dermatomyositis
76	M	Yes	ILD	Interstitial lung disease of unknown cause	Chronic autoimmune disease
72	M	Yes	UIP	Probable IPF	IPF
60	M	Yes	Probable UIP	Probable IPF	IPF
60	M	Yes	Probable UIP	Probable IPF	IPF
75	M	Yes	UIP	Probable IPF	IPF
68	M	Yes	Probable UIP	Probable IPF	IPF
69	M	Yes	UIP	IPF	IPF
54	M	Yes	ILD	ILD of unknown cause	IPF, very early case
82	M	Yes	UIP	IPF	IPF
69	M	Yes	Probable UIP	Probable IPF	IPF
74	M	Yes	UIP	IPF	IPF

### Immunohistochemistry

The tissues were either videothoracoscopic biopsies (17 from all autoimmune disease and some IPF patients and 6 cryobiopies from the remaining IPF patients). Serial sections were taken from selected tissue blocks. One slide was stained with hematoxylin-eosin, and further sections were incubated with antibodies for p16, p21, β-galactosidase, MAP1S, pAMPKα, LC3, SIRT1, TTF1, CK5/6, GLUT1, and LDH (detailed immunohistochemistry protocol is provided in Table 2).

**Table 2:** Immunohistochemistry of the antibodies and methodology. *MAP1S* microtubule-associated protein 1S, *pAMKα* phosphorylated adenosin-5′ monophosphate activated kinase a, *LC3* microtubule-associated protein 1A/1B-light chain 3, *SIRT1* silent information regulator T1, *TTF1* thyroid transcription factor 1, *CK* 5/6 cytokeratin 5/6, *GLUT1* glucose transporter type 1, *LDH* lactate-dehydrogenase

Antibody	P16 INK4A	P21	β-Galactosidase	MAP1s	PAMPKα	LC3	SIRT1	TTF1	CK 5/6	GLUT1	LDH
Producer	ENZO	DAKO	Abcam	Novus	Cell Signaling	Nano-tools	LSBio	DAKO	DAKO	Neo-markers	Abcam
Clone	ENZ-ABS-377-0100	SX118	Ab4761	NBP2-47383	THr172	0231-100/LC3-5F10	LS-B1895	M3575	B4rtu (DAKO GA780)	RB-9052-P	EP1566Y ab52488
Dilution	1:1000	1:30	1:1500	1:1000	1:100	1:200	1:500	1:100	Ready to use	1:200	1:100
Pretreatment	CC1 Ventana Ultra	MW9,0 (DAKO S2367)	CC1 Ventana Ultra	CC1 Ventana Ultra	CC1 Ventana Ultra	MW9,0 (DAKO S2367)	MW9,0 (DAKO S2367)	MW9,0 (DAKO S2367)	Flex high pH	MW Natrium-citrat 6,0	MW9,0 (DAKO S2367)
Detection kit	Ventana optiviev+ amplification	Envision DAKO K5007	Ventana optiviev + amplification	Ventana Ultra-View	Ventana Ultra-View	Envision DAKO K5007	Envision DAKO K5007	Envision DAKO K5007	HRP (GV800)	Envision DAKO K5007	Envision DAKO K5007

β-Galactosidase, p21, and p16 were used as markers for senescence. Autophagy was investigated by immunohistochemical staining for phosphorylated adenosin-5′ monophosphate activated kinase (pAMPKα), LC3, MAP1S, and SIRT1. In order to investigate the possible migration of circulating precursor cells of bronchiolar metaplasia cells, TTF1 was used as a surrogate marker for small airway epithelium and CK5/6 as marker for basal cells of the bronchiolar epithelium. Immunostaining for GLUT1 and LDH was done in order to quantify potential changes in cellular metabolism due to increased energy demand.

The presence (positive reaction) and absence of staining (negative reaction) with abovementioned antibodies was evaluated semiquantitatively for the following compartments: bronchial epithelium, airway-associated smooth muscle cells, vascular endothelium and smooth muscle cells, myofibroblasts, macrophages, bronchiolar metaplasia, and pneumocytes in unaffected lung areas and in areas of microcystic degeneration of lung parenchyma. Positivity was expressed as the percentage of analyzed cells for the markers of senescence (β-galactosidase, p21, and p16) and autophagy (pAMPKα, LC3, MAP1S, and SIRT1). The evaluation was done independently by two authors (HP, LB). In rare discrepant cases, slides were discussed on a multiheaded microscope and consensus was reached.

Of note, the term myofibroblast was preferred over fibroblasts, as these proliferating cells not only synthesize different collagens, but also contain myofilaments enabling them to migrate (they are stained less intense with smooth muscle actin compared with smooth muscle cells). Furthermore, the term cystic remodeling was preferred over honeycombing: the latter is a radiological term designating cystic structures in secondary lobules, while histopathologically remodeling can already be seen in primary lung lobules.

The study protocol was approved by the Ethics Committee of the Medical University of Graz (EK Number 24-135 ex 11/12).

## Results

### Study population

A total of 23 patients all with the histological pattern of UIP were investigated. Two groups were formed, one (*n* = 10) with UIP pattern and clinical diagnosis of IPF (UIP/IPF) and a second group (*n* = 13), for which clinical data pointed to an underlying immune disease (UIP/AuD). The age distribution was in the range of 52 to 82; the majority were man. All UIP/IPF patients were cigarette smokers, in contrast to 5/13 UIP/AuD patients. Based on clinical and laboratory data (not shown) as well as the histological findings, a final diagnosis could be established in all cases, although in some cases, a definite type of autoimmune disease was not possible (Table 1).

### Senescence

**Fig. 1:**
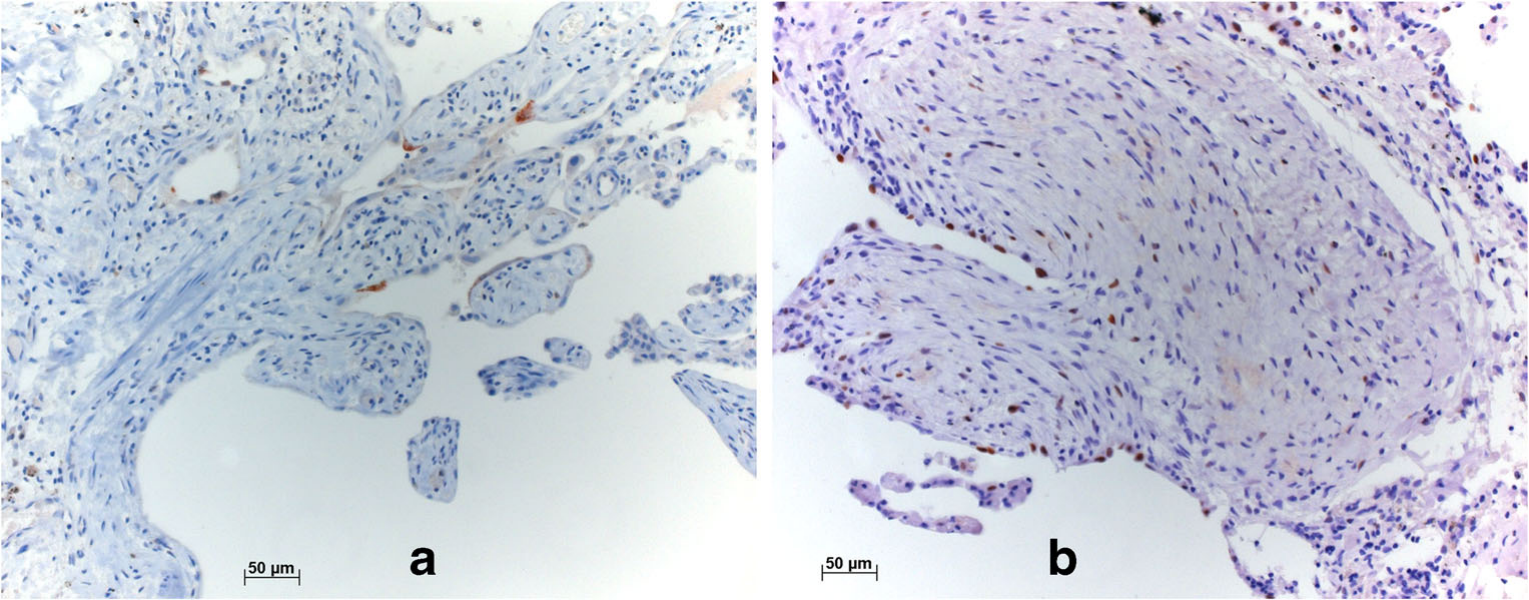
**a** Positive p16 reaction in senescent cells. **b** Presentation of staining with p21. Since the number of positive cells is high, this shows that p21 is expressed not only by senescent cells. Bars 50 μm

The staining pattern was evaluated separately for different compartments as stated previously. Epithelial cells (transformed pneumocytes and bronchiolar metaplasia) in remodeled areas were p16 and p21 positive in 22% and 57% of analyzed cases, respectively (Fig. 1a). Staining for p21 was positive in approximately 60% of normal pneumocytes as well (Fig. 1b), whereas p16 showed no positivity in normal pneumocytes. Neither p21 nor p16 was positive in myofibroblasts. A low percentage of normal bronchial/bronchiolar epithelial cells were positive for p21 in 19% of all cases, exclusively in UIP/AuD, whereas negative in UIP/IPF samples. p16 was negative in normal bronchial and bronchiolar epithelia in all cases. Endothelia, smooth muscle cells, and macrophages were negative for both p21 and p16. In other compartments, there was no difference concerning the expression of senescence markers in the two groups. Staining for β-galactosidase was not successful in formalin-fixed paraffin-embedded tissue despite several attempts with different protocols (Table 3).

**Table 3:** Detailed results of the markers for senescence and autophagy in UIP/IPF and UIP/AuD

		Degenerative/remodeled epithelium	Myofibroblasts	Normal pneumocytes	Bronchial epithelium	Endothelium	Smooth muscle cells	Macrophages
Senescence								
p21	UIP/IPF*	9/9	1/9	8/9	0/9	0/9	0/9	0/9
	UIP/AuD	13/13	0/13	13/13	6/13	0/13	0/13	0/13
p16	UIP/IPF*	9/9	0/9	1/9	0/9	0/9	0/9	0/9
	UIP/AuD	13/13	1/13	0/13	0/13	0/13	0/13	1/13
Autophagy								
SIRT1	UIP/IPF	10/10	7/10	7/10	6/10	0/10	0/10	10/10
	UIP/AuD	13/13	12/13	11/13	9/13	3/13	0/13	12/13
MAP1S	UIP/IPF	10/10	4/10	8/10	7/10	4/10	0/10	10/10
	UIP/AuD	13/13	6/13	7/13	7/13	2/13	0/13	12/13
LC3	UIP/IPF	10/10	10/10	10/10	10/10	9/10	0/10	10/10
	UIP/AuD	13/13	12/13	13/13	13/13	8/13	3/13	13/13
pAMPKα	UIP/IPF	10/10	9/10	9/10	10/10	2/10	2/10	10/10
	UIP/AuD	13/13	13/13	13/13	12/13	2/13	1/13	13/13

•In one case, the relevant lesions were not anymore present in some of the serial sections; therefore, this case was excluded from the evaluation

### Autophagy

**Fig. 2:**
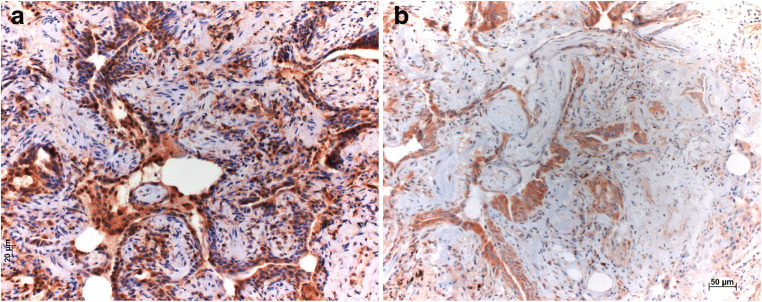
Autophagy markers LC3 (**a**) and phosphorylated AMPK (**b**) stain epithelial cells, including those in remodeled areas, but also myofibroblasts, Bars 50 μm

**Fig. 3:**
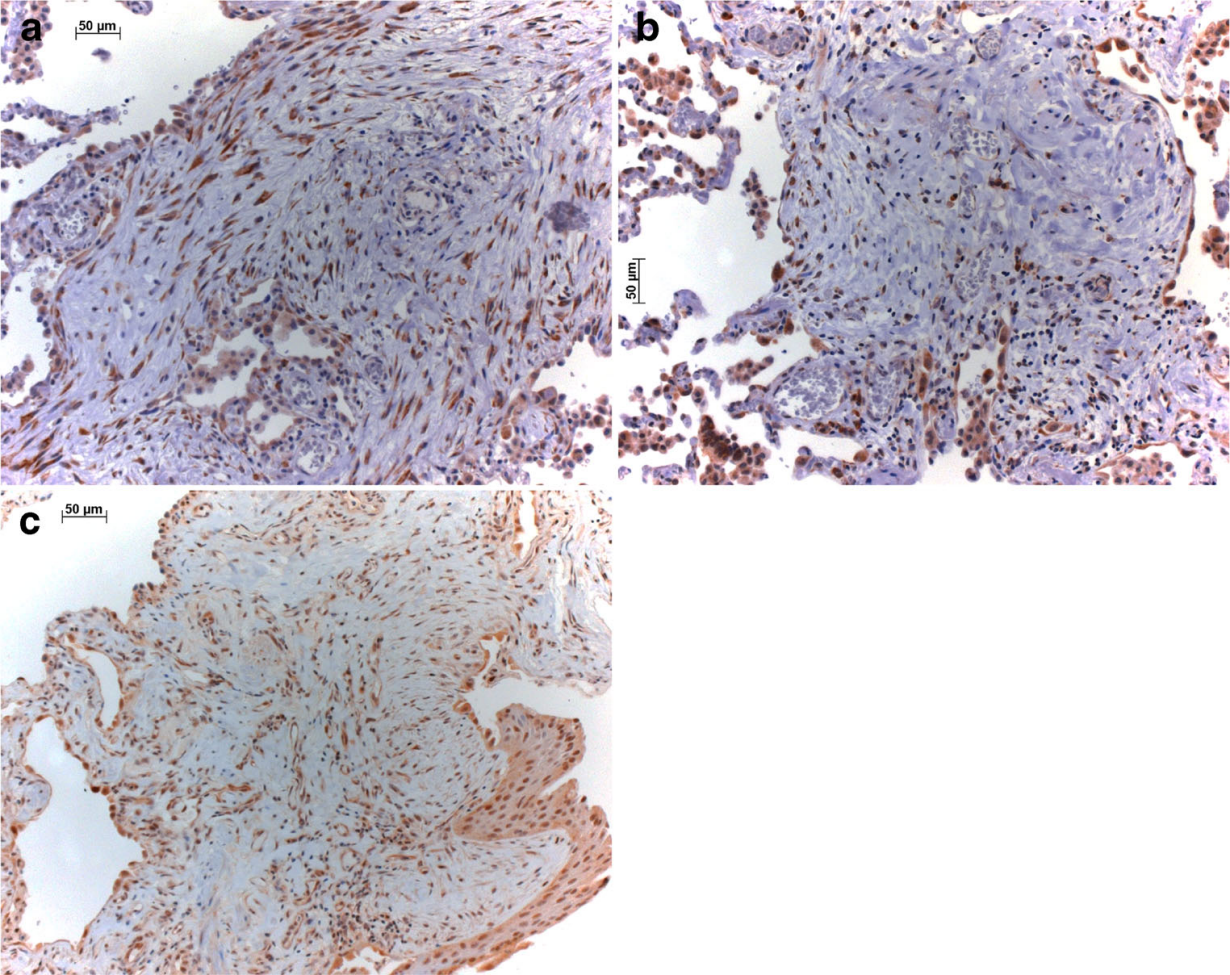
Autophagy markers SIRT in (**a**) young myofibroblasts, with a gradual loss of staining in older myofibroblasts and fibrocytes (**b**); MAP1S, another autophagy marker, is expressed in myofibroblasts and epithelial cells within remodeled areas (**c**). Bars 50 and 20 μm

Epithelial cells in remodeled areas were positive in more than 90% of all cases for SIRT1, MAP1S, LC3, and pAMKα (Table 3). Positive staining for LC3 (Fig. 2a) and pAMKα (Fig. 2b) was observed in normal pneumocytes in more than 90% of all cases, while SIRT1 (Fig. 3a, b) and MAP1S (Fig. 3c) were only occasionally seen in some normal pneumocytes. Myofibroblasts were LC3 and pAMKα positive in more than 90% of cases, whereas positive staining for SIRT1 and MAP1S was observed only in some cases.

More than 80% of cases demonstrated expression of LC3 and pAMKα in bronchial epithelia. SIRT1 and MAP1S, on the contrary, were only observed in some cases in bronchial epithelial cells. LC3 showed positivity in endothelia in most cases, whereas MAP1S, SIRT1, and pAMKα were observed in endothelial cells only in few cases. Positive staining for LC3 and pAMKα in smooth muscle cells was seen in few cases. SIRT1 and MAP1S were not observed in smooth muscle cells. Positive staining for SIRT1, MAP1S, LC3, and PAMKα was observed in alveolar macrophages in more than 60% of cases.

When comparing the staining pattern in UIP/IPF and UIP/AuD, there were no differences in the expression of autophagy markers (Table 3).

### Cellular metabolism with respect to hypoxia

**Fig. 4:**
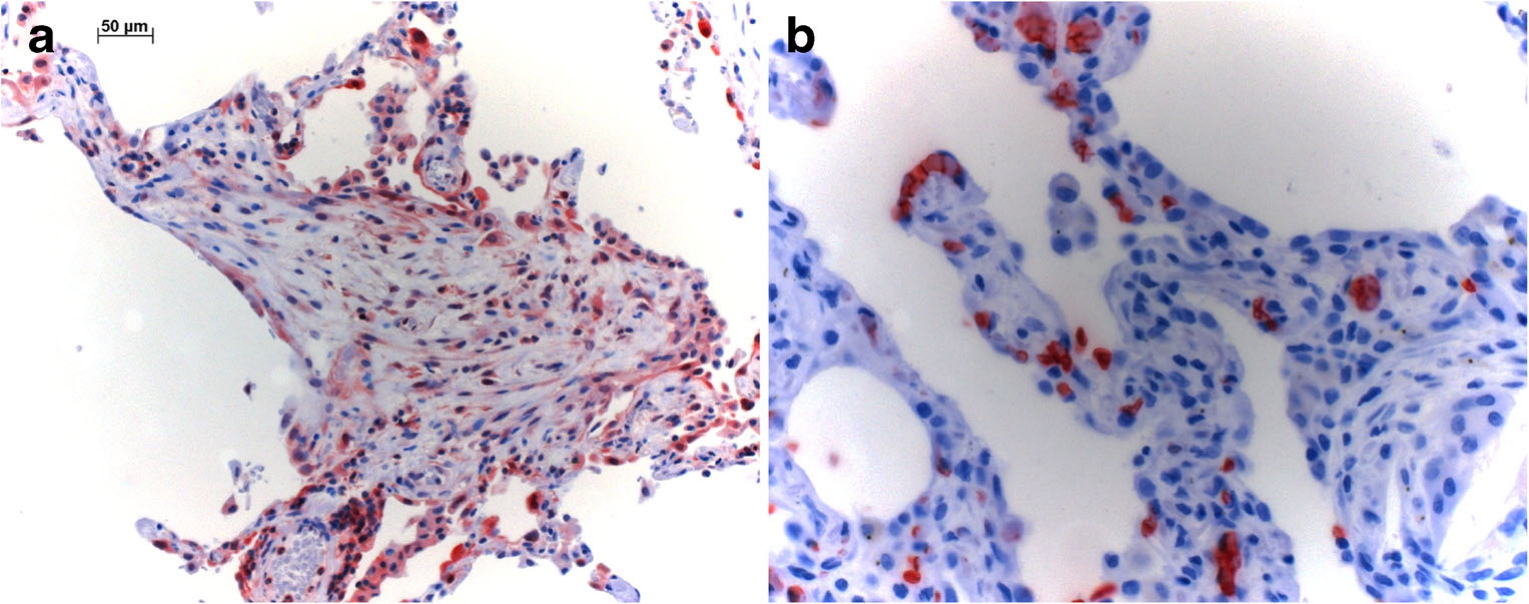
**a** Epithelial cells in remodeled areas, but also normal bronchial epithelia are positive for LDH; myofibroblasts also have upregulated this enzyme, but less intense. **b** Expression of GLUT1 is seen in red blood cells, but also in few epithelial cells within the remodeling area. Bar 50 μm and magnification × 400

Epithelial cells in remodeled areas stained positive for not only LDH, but also bronchial epithelia and macrophages (Fig. 4a). In few cases, LDH was expressed in endothelia as well, especially in larger vessels within remodeled areas. LDH was not expressed in pneumocytes in unaffected lung parenchyma, nor in myofibroblasts or smooth muscle cells. Positive staining for GLUT1 was observed in erythrocytes, epithelia in remodeled areas (Fig. 4b), and in bronchial epithelia of areas unaffected by fibrosis. The staining in cells of epithelia in remodeled areas was exclusively cytoplasmic and was not observed in normal pneumocytes. Staining for GLUT1 was absent in myofibroblasts, endothelia, smooth muscle cells, and macrophages. There was no difference with respect to UIP/IPF and UIP/AuD samples.

### Origin of epithelial cells

**Fig. 5:**
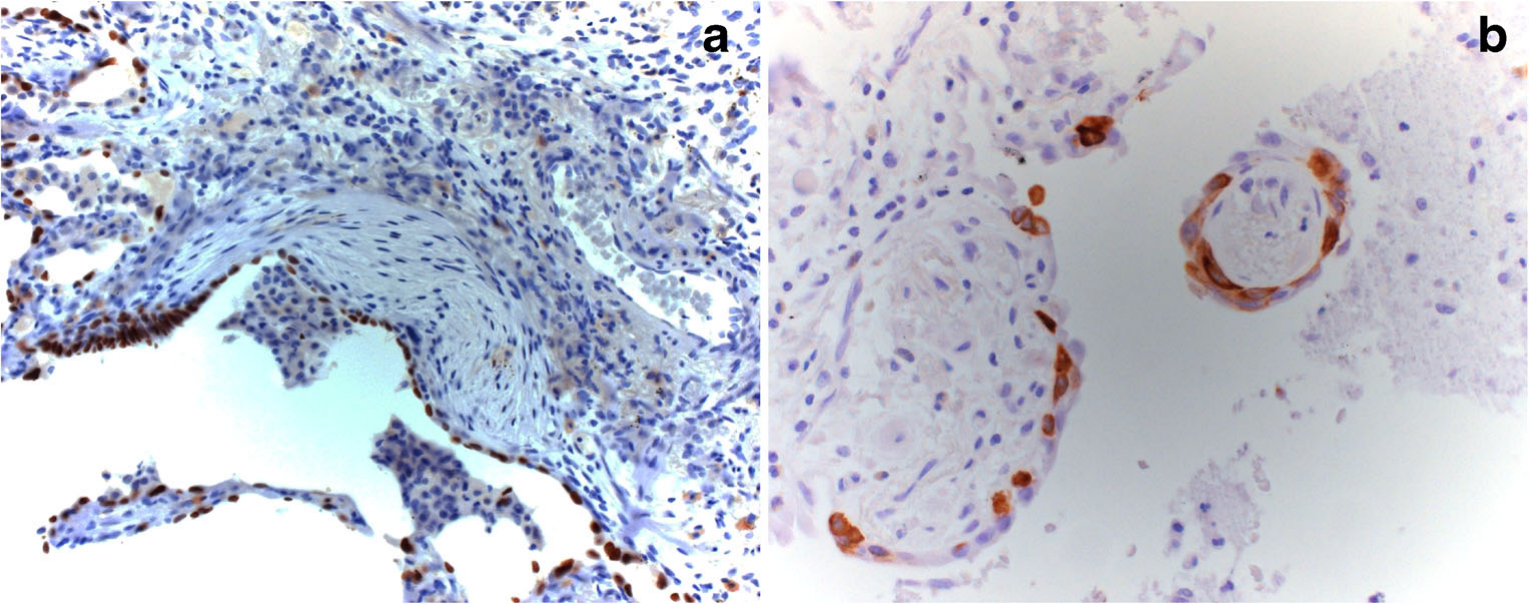
Epithelial cells in bronchioles and in regenerating cystic areas express TTF1 (**a**), a marker of the terminal bronchoalveolar unit as well as cytokeratin 5/6 (**b**), also found in basal cells of bronchioles and small bronchi. Magnification × 200 and × 400

Epithelial cells in remodeled areas were positive for both TTF1 and CK5/6 (Fig. 5a, b). Positive staining of CK5/6 was observed in basal cells in normal epithelium as well. Columnar and cuboidal cells of normal epithelium were negative. In addition, positivity was seen in squamous metaplasia within the remodeled areas. TTF1 showed positivity in normal epithelium as well as in pneumocytes and regenerating cells in remodeled areas. Myofibroblasts, smooth muscle cells, endothelium, and macrophages were all negative. Here as well, there was no difference between UIP/IPF and UIP/AuD samples.

## Discussion

Senescence has been identified as a strong factor contributing to fibrosis in the lung, kidney, and liver [2, 35, 45, 65]. In lung diseases, it is activated in UIP. Senescent cells release different inflammatory mediators, most prominently interleukin-1a, interleukin-1b, interleukin-6, interleukin-10, and TGF-β. This release causes a prolonged repair process, finally resulting in remodeling of the peripheral lung tissue and fibrosis [29]. Several markers of senescent cells have been identified, such as p16, p21, and β-galactosidase [19, 45]. In our study, p16 and p21 expression was present in remodeled areas of UIP, where p16 is much more specific for senescent cells, whereas p21 is expressed in additional cells, such as pneumocytes and normal bronchiolar cells. We did not succeed in staining for β-galactosidase in formalin-fixed paraffin-embedded tissues. Senescence plays a role in UIP regardless of the underlying etiology, as the expression of p16 and p21 was similar in UIP/IPF and UIP/AuD cases. Interestingly, myofibroblasts did not express p16 or p21; therefore, they do not undergo senescence. This implies that proliferation of myofibroblasts might be stimulated by growth factors released by the senescent epithelial cells.

The role of autophagy in UIP is controversially discussed. Some studies reported downregulation, others upregulation; however, some were experimental studies, and others conducted an examination of human UIP/IPF samples with immunohistochemical staining [5, 17, 37, 43]. Autophagy has two roles: one is to remove debris and downregulate inflammation and the other, frequently seen in cancer, is to phagocytose cellular debris, degrade it, and use it for metabolic purposes of the cells [50]. The second mechanism might be especially important in areas of hypoxia—a common phenomenon in cancer. In UIP, regardless of the underlying etiology, we found high expression of all four markers of autophagy (LC3, SIRT1, MAP1S, and pAMPKα) in epithelial cells within the remodeled areas and in myofibroblasts. Interestingly, expression was predominantly seen in myofibroblasts (“young lesions”), whereas fibrotic areas (“old lesions”) whwereereuse were instead of where matured and no longer proliferating fibrocytes were present, were less metabolic active. Upregulation of autophagy is here very likely the answer of epithelial cells and myofibroblasts to local hypoxia. This corresponds well with morphology: the thickness of alveolar septa is increased in these areas, and there is no neoangiogenesis (in contrast to organizing pneumonia); therefore, the cells have difficulties to support their metabolic needs. The expression of autophagy markers was also present in macrophages. This fits well into the picture, as they are within the alveolar lumina, have no access to nutrients from capillaries, and act very likely in the same way for their metabolic needs. Interestingly, hypoxia may also be responsible for the proliferation of metaplastic cells in the remodeled areas [59]. Very likely, hypoxia also induces the proliferation or differentiation of myofibroblasts [4].

We tried to tackle the question of hypoxia in remodeled areas using the expression of LDH and GLUT1. LDH is a marker for anaerobic glycolysis or the metabolic pathway of the Warburg type [43]. A positive staining was observed in cells within the remodeled areas, which can be interpreted as a sign of hypoxia. Whether anaerobic glycolysis takes place, because oxygen is scarce due to the increased demand and the impairment of vascularization through fibrosis, or because glycolysis takes place in exchange for oxidative phosphorylation (Warburg effect) as seen in neoplastic processes, needs further investigation [14]. Positive staining for GLUT1 in remodeled areas is another indicator for a high cellular energy demand, since GLUT1 expression reflects an increased cellular glucose uptake and glycolysis [10, 62]. Regarding the GLUT1 staining pattern, it must be noted that due to high-intensity staining of erythrocytes, the evaluation of the cells within remodeled areas and also unaffected lung tissue was difficult to determine.

There have been some speculations about the origin of cells within remodeled areas of the peripheral lung. As these cells sometimes differentiate into squamous cell metaplasia, an origin from large airways was discussed [48]. To contribute to this discussion, we performed immunohistochemistry using markers for peripheral cells. TTF1 and CK5/6 were expressed in epithelial cells in remodeled areas. CK5/6 was also seen in basal cells of the bronchioles. This could imply that cells from the bronchiolar epithelium move into the denuded alveolar region and repopulate these areas. The expression of TTF1 in these cells was a strong argument for their peripheral origin, as cells from bronchi do not express this protein.

## Conclusion

UIP regardless of the underlying etiology is driven by p16/p21-positive senescent cells within the remodeled area, which sustain the proliferation of myofibroblasts by inflammatory cytokines. Upregulation of autophagy in the setting of UIP seems to protect epithelia and myofibroblasts against hypoxia. In addition, the metabolism is changed to a glycolysis pathway demonstrated by the expression of LDH and GLUT1 within the remodeled foci. Because pneumocytes undergo apoptosis in areas of active remodeling (myofibroblastic foci), the epithelial surface layer is regenerated by basal cells from terminal bronchioles expressing TTF1 and CK5/6.

## Data Availability

All data are available within the manuscript. No additional data are deposited.
